# Supplementary material for: Immunogenicity and Safety of an Inactivated Quadrivalent Influenza Vaccine Administered Concomitantly with a 23-Valent Pneumococcal Polysaccharide Vaccine in Adults Aged 60 Years and Older
Source: Vaccines (Basel). 2024 Aug 22;12(8):935. doi: 10.3390/vaccines12080935 (PMC11360506; doi:10.3390/vaccines12080935)
Supplement: Supplementary file 1 [file vaccines-12-00935-s001.zip › vaccines-3086109-supplementary.pdf]

Supplementary materials

Contents:

1. Inclusion and exclusion criteria
2. Tables
Table S1. Demographic characteristics and health conditions of participants
Table S2. Pre-vaccination influenza HI antibody levels
Table S3. Post-vaccination influenza HI antibody levels
Table S4. Post-vaccination GMTs and GMT rations of S1 and S2 subgroups for influenza HI antibodies.
Table S5. Pre-vaccination antibody levels of 23 pneumococcal serotypes
Table S6. Post-vaccination antibody levels of 23 pneumococcal serotypes
Table S7. Post-vaccination GMCs and GMC rations of S1 and S2 subgroups for pneumococcal antibodies.

1 Inclusion and exclusion criteria

1.1 Inclusion criteria

Participant who:

(1) was 60 years of age or older;

(2) could understand and voluntarily sign the informed consent form;

(3) could provide valid legal proof of identity.

1.2 Exclusion criteria

Participant who:

(1) had already received a 2021-2022 seasonal influenza vaccine before screening;

(2) had received PPSV23 within the last five years;

(3) had a history of severe allergic reactions to vaccines;

(4) had uncontrolled epilepsy or other serious neurological disorders (such as transverse myelitis, Guillain-Barre syndrome and demyelinating disease);

(5) were experiencing a fever, an acute exacerbation of chronic diseases, uncontrolled severe chronic diseases or an acute illness;

(6) had received other investigational drugs within 30 days before vaccination with the test vaccine;

(7) had been received live attenuated vaccines within 14 days before vaccination with the test vaccine;

(8) had been received any subunit or inactivated vaccines within 7 days before vaccination with the test vaccine;

(9) had any other risk factors deemed unsuitable for participation by the investigator.

Table S1. Demographic characteristics and health conditions of participants

Characteristics		C Group (N=160)	S1 Subgroup (N=160)	S2 Subgroup (N=160)	P-value (3 groups)
Age, years *		68.36±5.30	68.44±5.30	68.97±5.40	0.5442
Age groups, years	60-69, n (%)	106(66.25%)	101(63.13)	91(56.88)	0.2073
	70-79, n (%)	49(30.63%)	54(33.75)	62(38.75)	
	80-89, n (%)	5(3.13%)	5(3.13)	7(4.38)	
Gender	Male, n (%)	74(46.25)	80(50.00)	74(46.25)	0.7403
	Female, n (%)	86(53.75)	80(50.00)	86(53.75)	
Ethnicity	Han, n (%)	160(100.00)	160(100.00)	160(100.00)	>0.999
Height (cm)*		162.15±8.37	161.93±8.22	160.94±8.92	0.3989
Weight (kg)*		65.91±11.41	65.96±11.18	64.59±9.75	0.4364
BMI*		24.98±3.27	25.10±3.50	24.92±3.08	0.8838

*Mean ± standard deviation. C group: co-administration group. S1 subgroup: separate administration subgroup 1; S2 subgroup: separate administration subgroup2.

Table S2. Pre-vaccination antibody levels of four influenza virus strains

Subtype		C Group (N=156)		S1 subgroup (N=151)		S2 subgroup (N=145)		P value	
		value	95%CI	value	95%CI	value	95%CI		
A/H1N1	SPR, %(n)	22.44 (35)	16.15-29.80	19.21 (29)	13.26-26.40	22.07 (32)	15.61-29.70	3 groups	0.7533
	GMT	12.71	10.70-15.10	11.58	9.71-13.82	12.76	10.60-15.36	3 groups	0.6901
A/H3N2	SPR, %(n)	82.05 (128)	75.11-87.73	83.44 (126)	76.54-88.99	84.83 (123)	77.93-90.24	3 groups	0.8111
	GMT	59.93	52.20-68.81	71.98	61.86-83.77	71.33	62.13-81.89	3 groups	0.1264
B/Victoria	SPR, %(n)	12.82 (20)	8.01-19.10	16.56 (25)	11.01-23.46	17.93 (26)	12.06-25.16	3 groups	0.4481
	GMT	13.53	12.01-15.24	13.23	11.56-15.15	12.88	11.19-14.84	3 groups	0.8746
B/Yamagata	SPR, %(n)	48.72 (76)	40.65-56.84	52.98 (80)	44.70-61.14	55.86 (81)	47.39-64.09	3 groups	0.4573
	GMT	28.92	25.74-32.49	31.51	27.96-35.50	37.41	32.45-43.12	3 groups	0.0157
								S1 vs S2	0.0676
								C vs S1	0.3109
								C vs S2	0.006

C group: concomitant administration group; S1 subgroup: separate administration subgroup 1; S2 subgroup: separate administration subgroup 2; SPR: seroprotection rate (⩾1:40); GMT: geometric mean titer.

Table S3. Post-vaccination SPRs, SCRs and GMFRs for influenza HI antibody against four virus strains

Subtype		C Group (N=156)		S1 subgroup (N=151)		S2 subgroup (N=145)		P value	
		value	95%CI	value	95%CI	value	95%CI		
A/H1N1	SPR, %(n)	91.67 (143)	86.17-95.49	94.04 (142)	88.99-97.24	91.03 (132)	85.16-95.14	3 groups	0.5912
	SCR, %(n)	89.74 (140)	83.88-94.02	92.72 (140)	87.34-96.31	90.34 (131)	84.33-94.62	3 groups	0.6345
	GMT	580.4	430.19-783.07	741.27	544.64-1008.88	832.46	610.47-1135.19		
	adjusted GMT	567.82	433.17-744.31	776.49	589.67-1022.50	812.11	613.32-1075.33	3 groups	0.1418
	GMFR	45.66	34.78-59.94	64.00	47.89-85.54	65.24	49.34-86.26	3 groups	0.1326
A/H3N2	SPR, %(n)	100.00 (156)	97.66-100.00	98.68 (149)	95.30-99.84	97.24 (141)	93.09-99.24	3 groups	0.0694
	SCR, %(n)	76.92 (120)	69.51-83.28	74.17 (112)	66.43-80.94	77.24 (112)	69.55-83.79	3 groups	0.7903
	GMT	499.02	412.14-604.21	605.7	498.40-736.10	541.4	432.37-677.93		
	adjusted GMT	513.45	421.72-625.12	596.02	488.17-727.70	533.93	435.56-654.53	3 groups	0.5587
	GMFR	8.33	6.74-10.28	8.41	6.73-10.52	7.59	5.91-9.75	3 groups	0.789
B/Victoria	SPR, %(n)	83.33 (130)	76.54-88.81	83.44 (126)	76.54-88.99	82.07 (119)	74.84-87.94	3 groups	0.9409
	SCR, %(n)	72.44 (113)	64.72-79.28	71.52 (108)	63.62-78.56	75.17 (109)	67.32-81.97	3 groups	0.7635
	GMT	92.63	75.64-113.45	98.81	80.55-121.21	92.34	73.96-115.28		
	adjusted GMT	91.51	75.43-111.02	98.76	81.15-120.19	93.61	76.60-114.38	3 groups	0.8563

	GMFR	6.85	5.65-8.29	7.47	6.05-9.22	7.17	5.76-8.93	3 groups	0.8398
B/Yamagata	SPR, %(n)	94.23 (147)	89.33-97.33	97.35 (147)	93.36-99.27	94.48 (137)	89.42-97.59	3 groups	0.3584
	SCR, %(n)	69.87 (109)	62.02-76.95	76.16 (115)	68.55-82.71	66.21 (96)	57.89-73.85	3 groups	0.1619
	GMT	156.48	130.85-187.14	202.21	168.95-242.00	161.54	134.02-194.71		
	adjusted GMT	163.31	137.50-193.96	204.2	171.55-243.06	152.72	127.72-182.61	3 groups	0.0557
	GMFR	5.41	4.58-6.40	6.42	5.31-7.76	4.32	4.77-5.95	3 groups	0.0168
								S1 vs S2	0.007
								C vs S1	0.1832
								C vs S2	0.1052

C group: concomitant administration group; S1 subgroup: separate administration subgroup 1; S2 subgroup: separate administration subgroup 2; SPR: seroprotection rate (⩾1:40); SCR: seroconversion rate; GMT: geometric mean titer; GMFR: geometric mean fold rise.

Table S4. Post-vaccination GMTs and GMT rations of S1 and S2 subgroups for influenza HI antibodies.

**Subtype**		**S1 subgroup N=151**	**S2 subgroup N=145**	**GMT ratio S2/S1**
H1N1	adjusted GMT	776.49	812.11	1.05
	95% CI	589.67,1022.50	613.32,1075.33	(0.71,1.55)
H3N2	adjusted GMT	596.02	533.93	0.9
	95% CI	488.17,727.70	435.56,654.53	(0.67,1.19)
BV	adjusted GMT	98.76	93.61	0.95
	95% CI	81.15,120.19	76.60,114.38	(0.72,1.25)
BY	adjusted GMT	204.2	152.72	0.75
	95% CI	171.55,243.06	127.72,182.61	(0.58,0.96)

S1 subgroup: separate administration subgroup 1; S2 subgroup: separate administration subgroup 2; GMT: geometric mean titer.

Table S5. Pre-vaccination antibody levels of 23 pneumococcal serotypes

Serotype		C Group (N=156)		S1 subgroup (N=142)		S2 subgroup (N=151)		P value	
		value	95%CI	value	95%CI	value	95%CI		
**1**	**GMC (ug/ml)**	1.12	0.97-1.28	1.24	1.07-1.44	1.09	0.94-1.25	3 groups	0.3908
**2**	**GMC (ug/ml)**	3.05	2.57-3.61	3.39	2.83-4.05	2.7	2.33-3.14	3 groups	0.1749
**3**	**GMC (ug/ml)**	0.45	0.38-0.52	0.46	0.40-0.52	0.43	0.37-0.50	3 groups	0.8538
**4**	**GMC (ug/ml)**	0.65	0.57-0.73	0.65	0.58-0.73	0.65	0.57-0.74	3 groups	0.9983
**5**	**GMC (ug/ml)**	0.45	0.39-0.51	0.49	0.43-0.56	0.46	0.41-0.53	3 groups	0.5620
**6B**	**GMC (ug/ml)**	1.19	0.99-1.42	1.2	0.98-1.47	1.36	1.14-1.61	3 groups	0.5331
**7F**	**GMC (ug/ml)**	1.14	0.99-1.30	1.39	1.21-1.61	1.26	1.08-1.47	3 groups	0.1408
**8**	**GMC (ug/ml)**	1.78	1.57-2.03	1.89	1.67-2.14	1.95	1.69-2.26	3 groups	0.6332
**9N**	**GMC (ug/ml)**	2.61	2.32-2.94	2.72	2.32-3.18	2.73	2.41-3.08	3 groups	0.8821
**9V**	**GMC (ug/ml)**	1.81	1.57-2.09	1.89	1.65-2.15	2.07	1.78-2.41	3 groups	0.4021
**10A**	**GMC (ug/ml)**	1.96	1.71-2.25	2.23	1.91-2.59	2.15	1.86-2.47	3 groups	0.4483
**11A**	**GMC (ug/ml)**	2.46	2.14-2.83	2.5	2.18-2.86	2.43	2.13-2.77	3 groups	0.9604
**12F**	**GMC (ug/ml)**	1.08	0.94-1.23	0.54	0.46-0.64	1.05	0.93-1.19	3 groups	<0.0001
								S1 vs S2	<0.0001
								C vs S2	0.785
								C vs S1	<0.0001
**14**	**GMC (ug/ml)**	4.79	4.22-5.44	4.74	3.95-5.68	5.72	4.97-6.58	3 groups	0.1428
**15B**	**GMC (ug/ml)**	3.93	3.43-4.51	4.47	3.83-5.21	4.97	4.33-5.70	3 groups	0.0674
**17F**	**GMC (ug/ml)**	1.18	1.00-1.39	1.29	1.07-1.56	1.33	1.14-1.56	3 groups	0.5579
**18C**	**GMC (ug/ml)**	2.04	1.82-2.30	2.04	1.80-2.31	2.18	1.95-2.44	3 groups	0.6722
**19A**	**GMC (ug/ml)**	4.92	4.40-5.51	5.08	4.53-5.70	4.99	4.55-5.47	3 groups	0.9189
**19F**	**GMC (ug/ml)**	2.07	1.82-2.35	1.99	1.72-2.30	1.99	1.76-2.26	3 groups	0.8997
**20**	**GMC (ug/ml)**	3.93	3.54-4.36	3.9	3.46-4.40	4.41	3.92-4.96	3 groups	0.2440
**22F**	**GMC (ug/ml)**	1.6	1.43-1.80	1.67	1.44-1.95	1.65	1.46-1.87	3 groups	0.8938
**23F**	**GMC (ug/ml)**	0.97	0.83-1.14	1.11	0.93-1.32	1.13	0.97-1.33	3 groups	0.3452
**33F**	**GMC (ug/ml)**	3.17	2.75-3.65	2.9	2.50-3.37	2.89	2.52-3.32	3 groups	0.5901

C group: concomitant administration group; S1 subgroup: separate administration subgroup 1; S2 subgroup: separate administration subgroup 2; GMC: geometric mean concentration.

Table S6. Post-vaccination antibody levels of 23 pneumococcal serotypes

Serotype		C Group (N=156)		S1 subgroup (N=142)		S2 subgroup (N=151)		P value	
		value	95%CI	value	95%CI	value	95%CI		
1	GMC	7.35	6.16,8.78	10.19	8.20,12.66	8.43	6.99,10.17		
	adjusted GMC	7.5	6.43,8.75	9.55	8.12,11.22	8.78	7.51,10.27	3 groups	0.0976
	GMFR	6.59	5.64-7.69	8.19	6.93-9.68	7.75	6.60-9.09	3 groups	0.1425
	2-fold increase rate, %(n)	90.38 (141)	84.64-94.52	92.96 (132)	87.43-96.57	90.73 (137)	84.93-94.84	3 groups	0.6981
2	GMC	20.4	17.21,24.18	25.6	21.07,31.10	20.19	17.11,23.83		
	adjusted GMC	20.31	17.70,23.30	23.83	20.62,27.53	21.69	18.86,24.96	3 groups	0.2887
	GMFR	6.7	5.70-7.86	7.56	6.50-8.79	7.47	6.45-8.65	3 groups	0.4702
	2-fold increase rate, %(n)	91.67 (143)	86.17-95.49	91.55 (130)	85.70-95.56	94.7 (143)	89.83-97.69	3 groups	0.4946
3	GMC	0.96	0.83,1.11	1.11	0.96,1.27	0.96	0.84,1.09		
	adjusted GMC	0.96	0.87,1.05	1.09	0.98,1.20	0.98	0.89,1.08	3 groups	0.1594
	GMFR	2.15	1.93-2.41	2.43	2.19-2.70	2.24	2.02-2.48	3 groups	0.2751
	2-fold increase rate, %(n)	43.59 (68)	35.68-51.75	57.04 (81)	48.47-65.31	47.02 (71)	38.86-55.30	3 groups	0.0567
4	GMC	2.57	2.21,2.98	3.01	2.59,3.49	2.92	2.49,3.44		
	adjusted GMC	2.57	2.28,2.90	3.01	2.65,3.41	2.92	2.58,3.30	3 groups	0.1694
	GMFR	3.96	3.47-4.51	4.62	4.09-5.22	4.48	3.94-5.10	3 groups	0.1944
	2-fold increase rate, %(n)	79.49 (124)	72.29-85.53	85.92 (122)	79.09-91.18	86.09 (130)	79.53-91.18	3 groups	0.2039
5	GMC	2.96	2.41,3.62	3.63	2.91,4.53	3.62	3.03,4.32		
	adjusted GMC	3.07	2.62,3.61	3.46	2.92,4.09	3.64	3.09,4.28	3 groups	0.3335
	GMFR	6.61	5.57-7.85	7.36	6.26-8.64	7.79	6.63-9.15	3 groups	0.3599
	2-fold increase rate, %(n)	87.82 (137)	81.64-92.51	91.55 (130)	85.70-95.56	91.39 (138)	85.73-95.34	3 groups	0.4644
6B	GMC	6.75	5.50,8.29	8.92	7.15,11.13	9.29	7.73,11.17		
	adjusted GMC	7.01	6.06,8.11	9.16	7.86,10.66	8.72	7.52,10.11	3 groups	0.0283
								C vs S2	0.0391
								C vs S1	0.013
								S1 vs S2	0.6528
	GMFR	5.69	4.88-6.64	7.41	6.38-8.60	6.85	5.82-8.07	3 groups	0.0527
	2-fold increase rate, %(n)	87.82 (137)	81.64-92.51	90.85 (129)	84.85-95.03	90.07 (136)	84.15-94.33	3 groups	0.6721
7F	GMC	8.32	7.00,9.90	9.55	7.86,11.60	9.65	8.04,11.59		
	adjusted GMC	8.98	7.78,10.37	8.82	7.58,10.25	9.61	8.31,11.12	3 groups	0.6895
	GMFR	7.32	6.34-8.46	6.85	5.92-7.93	7.65	6.51-8.98	3 groups	0.5981
	2-fold increase rate, %(n)	93.59 (146)	88.53-96.88	90.85 (129)	84.85-95.03	93.38 (141)	88.16-96.78	3 groups	0.6068

8	GMC	9.35	7.99,10.93	12.34	10.63,14.34	11.25	9.71,13.03		
	adjusted GMC	9.61	8.46,10.92	12.28	10.74,14.03	10.98	9.65,12.50	3 groups	0.0336
								C vs S2	0.1494
								C vs S1	0.0095
								S1 vs S2	0.2405
	GMFR	5.24	4.54-6.04	6.53	5.70-7.47	5.76	4.97-6.69	3 groups	0.0992
	2-fold increase rate, %(n)	86.54 (135)	80.16-91.47	93.66 (133)	88.31-97.06	89.4 (135)	83.36-93.82	3 groups	0.1266
9N	GMC	15.76	13.63,18.22	19.05	16.42,22.11	18.26	15.82,21.08		
	adjusted GMC	15.97	14.06,18.13	18.94	16.57,21.63	18.12	15.92,20.62	3 groups	0.1642
	GMFR	6.03	5.23-6.95	7.01	6.01-8.18	6.7	5.80-7.74	3 groups	0.3357
	2-fold increase rate, %(n)	87.82 (137)	81.64-92.51	89.44 (127)	83.18-93.97	89.4 (135)	83.36-93.82	3 groups	0.8767
9V	GMC	9.27	7.88,10.91	10.10	8.60,11.85	9.99	8.50,11.73		
	adjusted GMC	9.62	8.45,10.96	10.21	8.91,11.70	9.51	8.33,10.86	3 groups	0.7362
	GMFR	5.12	4.42-5.92	5.35	4.67-6.12	4.82	4.17-5.58	3 groups	0.5995
	2-fold increase rate, %(n)	85.9 (134)	79.43-90.95	88.73 (126)	82.35-93.42	82.78 (125)	75.80-88.43	3 groups	0.3456
10A	GMC	14.18	11.72,17.15	18.1	14.35,22.81	17.28	14.53,20.54		
	adjusted GMC	15.07	12.97,17.51	17.23	14.72,20.16	16.98	14.58,19.78	3 groups	0.4078
	GMFR	7.23	6.25-8.37	8.13	6.90-9.59	8.05	6.90-9.40	3 groups	0.4956
	2-fold increase rate, %(n)	91.67 (143)	86.17-95.49	91.55 (130)	85.70-95.56	91.39 (138)	85.73-95.34	3 groups	0.9962
11A	GMC	7.78	6.81,8.88	8.27	7.05,9.69	8.68	7.60,9.93		
	adjusted GMC	7.77	6.97,8.68	8.2	7.30,9.20	8.76	7.83,9.80	3 groups	0.327
	GMFR	3.16	2.81-3.56	3.31	2.94-3.73	3.58	3.13-4.09	3 groups	0.3575
	2-fold increase rate, %(n)	69.23 (108)	61.35-76.36	72.54 (103)	64.42-79.68	72.85 (110)	65.02-79.76	3 groups	0.7396
12F	GMC	3.43	3.01,3.91	2.93	2.32,3.70	3.68	3.22,4.20		
	adjusted GMC	3	2.60,3.46	3.86	3.31,4.52	3.26	2.82,3.78	3 groups	0.07
	GMFR	3.18	2.82-3.59	5.38	4.35-6.65	3.49	3.07-3.98	3 groups	<0.0001
								C vs S2	0.2957
								C vs S1	<0.0001
								S1 vs S2	0.0007
	2-fold increase rate, %(n)	70.51 (110)	62.69-77.53	84.51 (120)	77.49-90.03	72.85 (110)	65.02-79.76	3 groups	0.0114
								C vs S2	0.6499
								C vs S1	0.004
								S1 vs S2	0.0152
14	GMC	18.37	15.46,21.82	21.13	17.54,25.45	20.63	17.36,24.52		
	adjusted GMC	18.99	16.35,22.05	21.98	18.79,25.71	19.21	16.49,22.37	3 groups	0.3437
	GMFR	3.83	3.27-4.50	4.46	3.70-5.37	3.61	3.11-4.19	3 groups	0.1916

	2-fold increase rate, %(n)	66.67 (104)	58.68-74.00	76.06 (108)	68.18-82.81	66.23 (100)	58.09-73.71	3 groups	0.1204
15B	GMC	24.64	21.12,28.74	27.22	23.01,32.20	31.07	26.90,35.89		
	adjusted GMC	26.3	23.07,29.99	27.1	23.62,31.08	29.17	25.53,33.33	3 groups	0.5395
	GMFR	6.27	5.41-7.25	6.09	5.26-7.06	6.25	5.37-7.27	3 groups	0.9597
	2-fold increase rate, %(n)	87.82 (137)	81.64-92.51	88.73 (126)	82.35-93.42	87.42 (132)	81.05-92.25	3 groups	0.9395
17F	GMC	6.8	5.58,8.29	8.96	7.32,10.98	8.94	7.55,10.59		
	adjusted GMC	7.12	6.12,8.29	8.83	7.54,10.35	8.64	7.41,10.08	3 groups	0.1023
	GMFR	5.78	4.90-6.80	6.92	5.87-8.16	6.71	5.66-7.95	3 groups	0.2627
	2-fold increase rate, %(n)	84.62 (132)	77.98-89.89	87.32 (124)	80.71-92.31	86.09 (130)	79.53-91.18	3 groups	0.7965
18C	GMC	7.4	6.39,8.56	8.13	7.05,9.38	7.46	6.64,8.39		
	adjusted GMC	7.5	6.72,8.37	8.25	7.35,9.25	7.26	6.49,8.12	3 groups	0.2722
	GMFR	3.62	3.19-4.11	3.98	3.56-4.46	3.42	3.04-3.86	3 groups	0.2183
	2-fold increase rate, %(n)	76.28 (119)	68.82-82.72	85.92 (122)	79.09-91.18	78.15 (118)	70.70-84.45	3 groups	0.0921
19A	GMC	12.74	11.00,14.76	15.64	13.39,18.27	14.74	12.85,16.91		
	adjusted GMC	12.89	11.48,14.47	15.43	13.67,17.42	14.75	13.11,16.59	3 groups	0.0877
	GMFR	2.59	2.29-2.92	3.08	2.74-3.46	2.95	2.62-3.33	3 groups	0.1073
	2-fold increase rate, %(n)	54.49 (85)	46.33-62.47	71.13 (101)	62.93-78.42	66.89 (101)	58.77-74.32	3 groups	0.0075
								C vs S2	0.0262
								C vs S1	0.0031
								S1 vs S2	0.4332
19F	GMC	9.43	8.02,11.09	10	8.29,12.06	10.96	9.24,13.01		
	adjusted GMC	9.26	8.07,10.62	10.09	8.74,11.66	11.08	9.63,12.74	3 groups	0.2008
	GMFR	4.56	3.92-5.31	5.02	4.36-5.78	5.51	4.79-6.33	3 groups	0.1844
	2-fold increase rate, %(n)	81.41 (127)	74.41-87.18	85.21 (121)	78.29-90.61	86.09 (130)	79.53-91.18	3 groups	0.4898
20	GMC	12.45	10.49,14.78	12.58	10.56,14.98	13.75	11.89,15.91		
	adjusted GMC	12.89	11.43,14.54	13.11	11.55,14.87	12.76	11.29,14.42	3 groups	0.9555
	GMFR	3.17	2.81-3.58	3.22	2.82-3.69	3.12	2.78-3.50	3 groups	0.9338
	2-fold increase rate, %(n)	66.67 (104)	58.68-74.00	66.9 (95)	58.52-74.56	69.54 (105)	61.53-76.76	3 groups	0.8392
22F	GMC	6.74	5.77,7.88	8.64	7.15,10.42	8.67	7.35,10.22		
	adjusted GMC	6.86	5.98,7.86	8.51	7.38,9.82	8.63	7.51,9.92	3 groups	0.0353
								C vs S2	0.0209
								C vs S1	0.0325
								S1 vs S2	0.8927
	GMFR	4.2	3.65-4.84	5.16	4.48-5.93	5.25	4.51-6.11	3 groups	0.0547
	2-fold increase rate, %(n)	76.28 (119)	68.82-82.72	89.44 (127)	83.18-93.97	86.75 (131)	80.29-91.72	3 groups	0.0044

								C vs S2	0.0183
								C vs S1	0.0028
								S1 vs S2	0.4794
23F	GMC	5.02	4.23,5.96	6.38	5.23,7.79	5.75	4.84,6.82		
	adjusted GMC	5.35	4.66,6.15	6.21	5.37,7.18	5.52	4.79,6.35	3 groups	0.3132
	GMFR	5.18	4.41-6.07	5.75	4.93-6.70	5.07	4.41-5.83	3 groups	0.4736
	2-fold increase rate, %(n)	85.9 (134)	79.43-90.95	84.51 (120)	77.49-90.03	86.75 (131)	80.29-91.72	3 groups	0.8577
33F	GMC	24.8	20.64,29.79	30.86	25.19,37.81	27.25	23.00,32.29		
	adjusted GMC	23.78	20.44,27.67	31.5	26.88,36.91	27.9	23.93,32.54	3 groups	0.0413
								C vs S2	0.1464
								C vs S1	0.0121
								S1 vs S2	0.2809
	GMFR	7.83	6.69-9.17	10.64	9.03-12.54	9.44	8.04-11.07	3 groups	0.0276
	2-fold increase rate, %(n)	91.67 (143)	86.17-95.49	95.77 (136)	91.03-98.43	92.72 (140)	87.34-96.31	3 groups	0.3422
								C vs S2	0.1017
								C vs S1	0.0082
								S1 vs S2	0.3016

C group: concomitant administration group; S1 subgroup: separate administration subgroup 1; S2 subgroup: separate administration subgroup 2; GMC: geometric mean concentration; GMFR: geometric mean fold rise.

Table S7. Post-vaccination GMCs and GMC rations of S1 and S2 subgroups for pneumococcal antibodies.

**Serotype**		**S1 subgroup N=142**	**S2 subgroup N=151**	**GMT ratio S1/S2**
1	adjusted GMC	9.55	8.78	1.09
	95% CI	8.12,11.22	7.51,10.27	0.87,1.36
2	adjusted GMC	23.83	21.69	1.1
	95% CI	20.62,27.53	18.86,24.96	0.90,1.34
3	adjusted GMC	1.09	0.98	1.11
	95% CI	0.98,1.20	0.89,1.08	0.96,1.27
4	adjusted GMC	3.01	2.92	1.03
	95% CI	2.65,3.41	2.58,3.30	0.86,1.23
5	adjusted GMC	3.46	3.64	0.95
	95% CI	2.92,4.09	3.09,4.28	0.75,1.20
6B	adjusted GMC	9.16	8.72	1.05
	95% CI	7.86,10.66	7.52,10.11	0.85,1.30
7F	adjusted GMC	8.82	9.61	0.92
	95% CI	7.58,10.25	8.31,11.12	0.74,1.13
8	adjusted GMC	12.28	10.98	1.12
	95% CI	10.74,14.03	9.65,12.50	0.93,1.35
9N	adjusted GMC	18.94	18.12	1.05
	95% CI	16.57,21.63	15.92,20.62	0.87,1.26
9V	adjusted GMC	10.21	9.51	1.07
	95% CI	8.91,11.70	8.33,10.86	0.89-1.30
10A	adjusted GMC	17.23	16.98	1.01
	95% CI	14.72,20.16	14.58,19.78	0.82,1.26
11A	adjusted GMC	8.2	8.76	0.94
	95% CI	7.30,9.20	7.83,9.80	0.80,1.10
12F	adjusted GMC	3.86	3.26	1.18
	95% CI	3.31,4.52	2.82,3.78	0.95,1.47
14	adjusted GMC	21.98	19.21	1.14
	95% CI	18.79,25.71	16.49,22.37	0.92,1.42
15B	adjusted GMC	27.1	29.17	0.93
	95% CI	23.62,31.08	25.53,33.33	0.77,1.12
17F	adjusted GMC	8.83	8.64	1.02
	95% CI	7.54,10.35	7.41,10.08	0.82,1.27
18C	adjusted GMC	8.25	7.26	1.14
	95% CI	7.35,9.25	6.49,8.12	0.97,1.33
19A	adjusted GMC	15.43	14.75	1.05
	95% CI	13.67,17.42	13.11,16.59	0.88,1.24
19F	adjusted GMC	10.09	11.08	0.91
	95% CI	8.74,11.66	9.63,12.74	0.75,1.11
20	adjusted GMC	13.11	12.76	1.03
	95% CI	11.55,14.87	11.29,14.42	0.86,1.23

22F	adjusted GMC	8.51	8.63	0.99
	95% CI	7.38,9.82	7.51,9.92	0.81,1.20
23F	adjusted GMC	6.21	5.52	1.13
	95% CI	5.37,7.18	4.79,6.35	0.92,1.38
33F	adjusted GMC	31.5	27.9	1.13
	95% CI	26.88,36.91	23.93,32.54	0.91,1.41

S1 subgroup: separate administration subgroup 1; S2 subgroup: separate administration subgroup 2; GMC: geometric mean concentration.
